# Community-based self-management of chronic low back pain in a rural African primary care setting: a feasibility study

**DOI:** 10.1017/S1463423619000070

**Published:** 2019-04-29

**Authors:** Chinonso N Igwesi-Chidobe, Emma L Godfrey, Sheila Kitchen, Chika N Onwasigwe, Isaac O Sorinola

**Affiliations:** 1 Department of Medical Rehabilitation, Faculty of Health Sciences and Technology, College of Medicine, University of Nigeria (Enugu Campus), Enugu, Nigeria; 2 Department of Physiotherapy, School of Population Health Sciences, Faculty of Life Sciences and Medicine, King’s College London, London, UK; 3 Department of Psychology, Institute of Psychiatry, Psychology and Neuroscience, King’s College London, London, UK; 4 Department of Community Medicine, Faculty of Medical Sciences, College of Medicine, University of Nigeria (Enugu Campus), Enugu, Nigeria

**Keywords:** chronic low back pain, cognitive behavioural therapy, self-management

## Abstract

A small pragmatic non-randomised controlled study investigated the feasibility and acceptability of a novel theory-informed community-based self-management programme targeting the biopsychosocial factors associated with chronic low back pain disability in a rural Nigerian primary care centre. Participants either received the programme, once weekly for 6 weeks, or usual care. The programme combined group exercise sessions with group/individual discussion sessions, informed by cognitive behavioural therapy and motivational interviewing. Recruitment rate, intervention delivery, proportion of planned treatment attended, retention/dropout rate, adherence to recommended self-management strategies and biopsychosocial outcomes were used to determine feasibility. Structured qualitative exit feedback interviews ascertained acceptability. Recruitment rate was 100%, treatment uptake was 83% and loss to follow-up was 8%. Greater benefits for the self-management group compared with control were observed for primary and secondary biopsychosocial outcomes. Although the programme appears promising, the exploratory design of this study warrants more rigorous intervention testing following suggested programme improvement.

## Introduction

The lack of effective management of chronic low back pain (LBP) despite its significant burden in rural Nigeria [(70–85% one-year prevalence rate (Birabi *et al*., 2012)] increases disability and reinforces rural–urban disparity (Abdulraheem, 2007; Igwesi-Chidobe, 2012). Chronic LBP increases the risk of all-cause and cardiovascular mortality (Hoy *et al*., 2010). This could be linked to associations between disability and exercise incapacity (Dean and Söderlund, 2015); pain and increased psychosocial stress (Truchon, 2001); long-term use of opioids for chronic pain management and increased cardiovascular risk (Carragee, 2005). This is particularly relevant because people with chronic LBP habitually depend on opioids, which can be obtained over the counter without a doctor’s prescription in rural Nigeria (Igwesi-Chidobe *et al*., 2017b).

Psychosocial factors, such as fear avoidance beliefs, and occupational biomechanical factors, particularly heavy lifting and prolonged trunk bending, are associated with work-related disability, and increased LBP symptoms (Steenstra *et al*., 2005; McNee *et al*., 2011), as found in rural Nigeria (Igwesi-Chidobe *et al*., 2017b). Psychosocial factors including illness perceptions and fear avoidance beliefs, but not biomechanical factors are the predictors of functional disability in patients with chronic LBP in rural Nigeria (Igwesi-Chidobe *et al*., 2017a). In contrast, biomechanical factors are primarily targeted with no acknowledgement of psychosocial factors in the management of chronic LBP in rural Nigeria (Igwesi-Chidobe *et al*., 2015).

Limited access to conventional healthcare in rural Nigeria implies that self-management may be particularly useful because of its cost effectiveness and ease of access through community-based programmes. Self-management is ‘an individual’s ability to manage the symptoms, treatment, physical and psychosocial consequences, and lifestyle changes inherent in living with a chronic condition’ (Barlow *et al*., 2002). Evidence-based treatment guidelines for chronic LBP recommend providing advice and education to promote self-management, combined with physical and psychosocial management which includes exercise and cognitive behavioural therapy (CBT), for people with substantial disability (NICE, 2009).

Low literacy and motivation pose barriers to participation in CBT programmes (Ehde *et al*., 2014). However, integrating motivational interviewing with CBT may improve participation in populations with low motivation and literacy (Barrowclough *et al*., 2010; Jones *et al*., 2011) such as rural Nigeria. Postural training may be an additional requirement for people in rural Nigeria as they are mostly involved in manual work, possibly implicating occupational biomechanical factors. Evidence suggests that integrated interventions targeting posture, exercises and psychosocial factors improve return to work outcomes (Heymans *et al*., 2005). This study is aimed at assessing the feasibility and acceptability of a novel evidence-based theory-informed self-management programme – The ‘Good Back’ programme; and will primarily inform future larger scale process and outcome evaluations. This paper is reported according to the extended guidelines for pilot and feasibility studies (Thabane *et al*., 2016; Eldridge *et al*., 2016).

## Methods

### Study design

This study is an exploratory pragmatic non-randomised controlled feasibility study incorporating qualitative individual exit feedback interviews.

### Study setting/context

Primary health care is often the only conventional health care accessible to rural Nigerian dwellers. Therefore, this study took place in a rural primary care centre serving about 15 000 typical rural Nigerian dwellers in Enugu state of south-eastern Nigeria. Pharmacological interventions are predominant in Nigerian primary care centres and typically include immunisations, and management of acute infections such as malaria, typhoid, diarrhoea and common cold. Chronic non-communicable diseases, responsible for over 50% of the total adult disease burden and morbidity/mortality in rural Nigeria (Abegunde *et al*., 2007), are minimally targeted in primary care.

### Participant recruitment

Two village-wide announcements invited potential participants to the primary care centre where they were given information sheets and a detailed oral explanation of the study. They were given two days to decide on participation. Eligibility was ascertained via screening. Informed consent was subsequently obtained.

### Screening

Body charts were used to identify pain in the lower back. Screening questions were interviewer-administered to rule out the ‘red flags’ for LBP by excluding chronic LBP associated with underlying serious pathology, radiculopathy or spinal stenosis (Downie *et al*., 2013). Participants were aged 18 years and above, with pain lasting for more than 12 weeks.

### Intervention

The ‘Good Back’ programme is an evidence-based theory-informed community-based self-management programme for people with chronic LBP in rural Nigeria. The programme targeted maladaptive illness perceptions and behaviours, and fear avoidance beliefs – the most important predictors of self-reported and performance-based disability in rural Nigeria (Igwesi-Chidobe *et al*., 2017a; 2017b). The programme is theoretically underpinned by the self-regulatory model of illness cognitions (Leventhal *et al*., 1998). CBT techniques were used to challenge maladaptive back pain beliefs, emotions and behaviours – particularly drug dependence and cure seeking (Igwesi-Chidobe *et al*., 2017b). Any exercise improves pain-related functional disability, and postural hygiene may improve pain-related work disability (Liddle *et al*., 2004; Van Middelkoop *et al*., 2011). Motivational interviewing techniques were used to communicate health information, and facilitate exercise and posture-related behaviour change, in line with the systematic review findings (Igwesi-Chidobe *et al*., 2018). Exercises included aerobic, strengthening, neuromuscular, flexibility and relaxation exercises. Postural hygiene was demonstrated with culturally relevant functional activities, including farming, carpentry, lifting heavy objects, fetching water, sweeping etc. Illustration-only patient booklets were used to promote educational aspects of the programme in this population with about 40% illiteracy rate (Igwesi-Chidobe *et al*., 2017c; 2017b).

The programme is a six-week self-management programme delivered once weekly. The programme was mainly group-based. Inclusion of individual discussion sessions depended on participants’ demands for more intimate topics, particularly the impact of chronic LBP on sexuality. The six discussion themes were: challenging a biomechanical model of chronic LBP; challenging an infective-degenerative understanding of chronic LBP; challenging other negative thoughts about back pain; managing exercise, pacing, goal setting and relaxation; chronic disease and chronic pain; and managing/coping with flare ups, relaxation, help seeking and self-management. These themes were informed by previous qualitative studies in this population (Igwesi-Chidobe *et al*., 2015; 2017b) and back pain rehabilitation programmes. Each weekly session was based on a different theme.

Each programme session has six phases, including education about back pain and health care; mapping of existing illness perceptions; challenging maladaptive illness perceptions; formulation of alternative illness perceptions and associated behaviours; practising more adaptive behaviour, like exercise and postural hygiene, in a supervised session, and exploring the incorporation of these into daily lives; and testing and strengthening any alternative illness perceptions by confirming their utility in daily life.

### Assignment to study arms

Random allocation was not done in this exploratory study. Although this limited internal validity, convenient assignment ensured that (1) the few interested younger adults, male participants and non-farmers were purposively assigned equally into the study arms; (2) non-participation was reduced since this was the first non-pharmacological behaviour change intervention in this population with an entrenched pharmacological treatment model (Igwesi-Chidobe *et al*., 2017b). Moreover, this study was not aimed at establishing causal relationships.

### Outcome assessment

Pre- and post-test outcome assessments were done by a trained physiotherapist unaware of group assignment.

### Primary outcomes

Feasibility was assessed in terms of recruitment rate, intervention delivery, proportion of planned treatment attended, retention/dropout rate, adherence to recommended self-management strategies and the primary outcome of disability measured with the Igbo Roland Morris Disability Questionnaire (RMDQ) (Igwesi-Chidobe *et al*., 2017c). A total score of 24 signifies the highest possible disability and 0 means no disability. Adherence to recommended exercises was assessed with the Exercise Adherence Rating Scale (EARS) (Newman-Beinart *et al*., 2017). A maximum score of 24 signifies perfect adherence and lower values reflect poorer adherence.

Acceptability of the programme was ascertained for all participants in the self-management group, using structured qualitative exit feedback interviews. An open-ended Igbo interview guide explored participants’ experiences of the programme, adherence behaviour and suggestions for programme improvement. Interviews were recorded verbatim as text.

### Secondary outcomes

These were performance-based disability [(Back Performance Scale; Strand *et al*., 2002)], illness perceptions [(Brief Illness Perception Questionnaire – BIPQ (Broadbent *et al*., 2006)], fear avoidance beliefs [(Fear Avoidance Beliefs Questionnaire – FABQ (Waddell *et al*., 1993)], pain intensity [(11-point box scale – 11-BS (Hawker *et al*., 2011)], pain medication use, systolic and diastolic blood pressure. Pain medication use was measured by determining the number of pain tablets ingested in the past two weeks due to back pain. Blood pressure was measured using a mercury sphygmomanometer as an exercise precaution.

All measures except performance-based disability and blood pressure were self-reported, and hence were interviewer-administered using cross-culturally adapted measures (Beaton *et al*., 2000).

### Timing of outcome assessment

Recruitment rate, reflection on intervention delivery, proportion of planned treatment attended and retention/dropout rate were assessed while the programme was ongoing. All primary and secondary outcomes except exercise adherence were administered at baseline and immediately after the programme. Exercise adherence for a past week was assessed at the beginning of each programme session.

### Sample size

Ten was an adequate size for one self-management group, in line with the Stanford self-management support approach and the National Institute for Health and Clinical Excellence (NICE) guideline recommendations (NICE, 2009; Lawn and Schoo, 2010). This study was not designed to confirm efficacy or effectiveness.

### A priori feasibility criteria

An acceptable effect size was set at 0.2 in self-reported disability – RMDQ, in line with the self-management literature (Warsi *et al*., 2003; Du *et al*., 2011). Based on feasibility criteria for CBT interventions (Pincus *et al*., 2013; Pincus *et al*., 2015), this study aimed at achieving at least 50% recruitment rate, 60% programme completion, and 85% programme attendance for one session. Loss of data was set at not exceeding 35% (10% due to non-compliance and 25% loss to follow-up).

### Intervention delivery

The lead author, a physiotherapist with 15 years of clinical experience in primary care and community-based rehabilitation, with some training in CBT and motivational interviewing, delivered the intervention. Each programme session lasted approximately 2 h with additional 30 min of break periods.

### Data analyses

Quantitative data analyses were mainly descriptive. Proportions/percentages, means and standard deviations of pre- and post-test outcomes (within-group data) and change scores (between-group data) were calculated using SPSS version 22. Effect sizes (between-group) were calculated with Hedges’ g and Glass’s Δ (Lakens, 2013).

Qualitative inductive content analysis reflecting a quantitative analysis of meaning (number of people reporting a theme) was performed with NVivo version 10. Interview transcripts were translated to English using evidence-based guidelines (Chen and Boore, 2010). Analysis using a manifest rather than interpreted content of interview transcripts was done due to the structured interview format that directly answered specific questions for programme improvement.

## Results

### Participants

**Figure 1:**
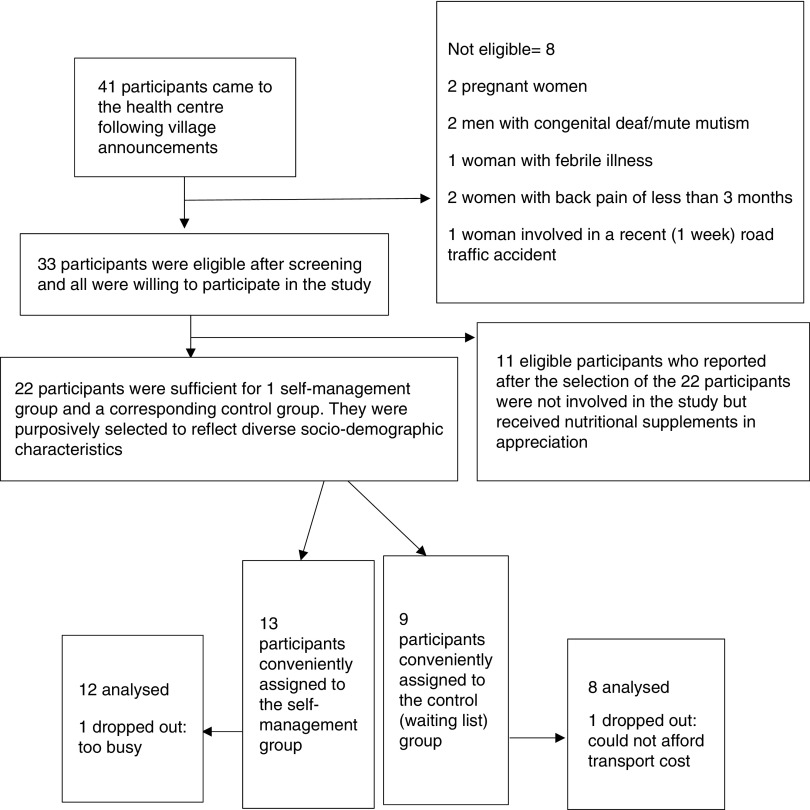
Recruitment process

Figure 1 details participant selection process. A total of 13 participants willing to come for the weekly sessions at the primary care centre were conveniently assigned to the Good Back programme. Nine participants that only wanted to come once were assigned to the control group.

### Baseline data

Table 1 shows that self-reported disability (RMDQ), fear avoidance beliefs (FABQ), systolic and diastolic blood pressure were balanced in the two groups.

**Table 1:** Demographic and clinical baseline characteristics by study arm

Variables	Good Back programme *n*=13	Usual care (waiting list) *n*=9
Gender: frequency (%)		
Female	10 (76.9)	6 (66.7)
Male	3 (23.1)	3 (33.3)
Age (years): mean (SD)	53.9 (14.1)	60.3 (13.6)
Education (years): mean (SD)	5.3 (5.4)	4.4 (4.7)
Marital status: frequency (%)		
Married	7 (53.8)	5 (55.6)
Widowed	5 (38.5)	4 (44.4)
Single	1 (7.7)	0 (0)
Occupation: frequency (%)		
Self-employed trading/farming	8 (61.5)	6 (66.7)
Unemployed (health reasons)	3 (23.1)	2 (22.2)
Student	1 (7.7)	0 (0)
Retired	1 (7.7)	1 (11.1)
Religion: frequency (%)		
Catholic	7 (53.8)	6 (66.7)
Anglican	3 (23.1)	0 (0)
Methodist	2 (15.4)	0 (0)
Pentecostal	1 (7.7)	3 (33.3)
Co-morbidity: frequency (%)		
None	5 (38.5)	1 (11.1)
HBP	5 (38.5)	6 (66.7)
Diabetes	1 (7.7)	0 (0)
Headache+toothache	1 (7.7)	1 (11.1)
Eye problems	1 (7.7)	1 (11.1)
LBP duration (years): mean (SD)	6.8 (4.1)	9.3 (15.4)
Systolic blood pressure (mmHg): mean (SD)	130.5 (23.5)	130.0 (32.2)
Diastolic blood pressure (mmHg): mean (SD)	80.7 (9.5)	75.0 (10.5)
Pain tablets in the last 2 weeks: mean (SD)	17.3 (14.0)	28.2 (34.2)
RMDQ: mean (SD)	17.0 (5.8)	17.8 (4.6)
Pain intensity (Igbo-11-BS): mean (SD)	6.8 (1.7)	5.3 (1.6)
BIPQ: mean (SD)	34.9 (10.7)	41.1 (9.7)
FABQ: mean (SD)	64.0 (21.9)	65.3 (15.8)
BPS: mean (SD)	3.2 (2.1)	4.6 (2.7)

SD: standard deviation

### Intervention fidelity

A multi-disciplinary research team appraised the video recordings of all six sessions to confirm intervention fidelity.

### Proportion of planned treatment attended

Programme attendance rate was 83%. The proportion of participants with 100% attendance was 77%. Of the three males in the self-management group, one attended only half (before phase five) of one session out of the six sessions. Another male participant came late for two sessions (after phase four and during phase one). The third male left after phase four in one session was late during phase four in one session, and came late during phase one of another session.

### Retention/dropout rate

Retention rate for the self-management programme was 92%. Dropout rate (loss to follow-up) was 8% in the self-management group and 11% in the usual care/waiting list group (Figure 1).

### Adherence to recommended self-management strategies

Exercise adherence (EARS) increased with the first few sessions of the programme, reduced to the starting values in the mid sessions and then increased beyond the starting values with subsequent sessions: 15.9 (5.2); 17.9 (4.5); 18.4 (4.9); 16.6 (6.0); 20.6 (3.7) and 20.5 (2.9).

### Outcomes and estimation

Table 2 shows that there were better improvements in all outcomes in the self-management group. Table 3 depicts mean baseline and change scores split by gender which showed that gender-related programme attendance influenced outcomes.

**Table 2:** Means and effect sizes in the self-management and usual care/waiting list groups

	Post-test mean values (SD)		Within-group/pre- and post-test mean differences (SD)		95% Confidence intervals of within-group mean differences				
Variables	Good Back programme *n*=12	Usual care (waiting list) *n*=8	Good Back programme *n*=12	Usual care (waiting list) *n*=8	Good Back programme *n*=12	Usual care (waiting list) *n*=8	Between-group mean differences	95% Confidence intervals of between-group differences	Between-group effect sizes Hedges’ g (Glass’s Δ)
RMDQ	7.0 (4.2)	19.6 (5.6)	−9.8 (4.8)	1.5 (3.9)	−12.8, −6.7	−1.8, 4.8	−12.6	−17.2, −8.0	−2.5 (−2.2)
BPS	2.1 (1.6)	4.6 (2.7)	−0.8 (1.3)	0.3 (2.8)	−1.6, −0.03	−2.1, 2.6	−2.5	−4.5, −0.5	−1.2 (−1.0)
BIPQ	13.5 (9.5)	45.6 (15.1)	−21.6 (15.2)	3.8 (7.5)	−31.3, −11.9	−2.5, 10.0	−32.1	−43.6, −20.6	−2.6 (−2.1)
FABQ	20.3 (15.9)	73.5 (28.5)	−41.7 (27.6)	7.8 (35.8)	−59.2, −24.1	−22.2, 37.7	−53.2	−74.0, −32.4	−2.3 (−1.9)
11-BS	2.8 (1.3)	7.0 (2.5)	−3.8 (2.1)	1.5 (3.1)	−5.2, −2.5	−1.1, 4.1	−4.2	−5.9, −2.4	−2.2 (−1.7)
Number of Pain tablets	3.2 (5.0)	28.5 (37.8)	−14.6 (12.1)	−2.8 (20.3)	−22.3, −6.9	−19.7, 14.2	−25.3	−48.3, −2.4	−1.0 (−0.7)
SBP (mmHg)	118.3 (11.6)	131.3 (30.0)	−12.5 (23.7)	2.0 (35.6)	−29.5, 4.5	−42.2, 46.2	−13.0	−32.9, 6.9	−0.6 (−0.4)
DBP (mmHg)	70.8 (13.3)	75.6 (15.0)	−5.8 (14.4)	0.0 (10.0)	−16.1, 4.5	−12.4, 12.4	−4.8	−18.2, 8.6	−0.3 (−0.3)

SBP= systolic blood pressure; DBP= diastolic blood pressure

**Table 3:** Baseline outcome scores and mean changes by gender in the self-management group

	Males *n*=3			Females *n*=9		
Variables	Mean baseline values (SD)	Pre–post-test mean changes (SD)	95% Confidence intervals	Mean baseline values (SD)	Pre–post-test mean changes (SD)	95% Confidence intervals
RMDQ	14.5 (5.5)	−1.8 (7.2)	−9.4, 5.7	18.4 (4.9)	−6.7 (6.8)	−10.7, -2.8
BPS	3.3 (2.9)	0.7 (2.8)	−2.3, 3.6	3.9 (2.2)	−0.9 (1.5)	−1.7, -0.01
BIPQ	31.8 (8.9)	−3.7 (10.2)	−14.3, 7.0	39.5 (10.6)	−14.8 (19.6)	−26.1, -3.5
FABQ	52.7 (19.2)	−10.8 (34.7)	−47.3, 25.6	69.0 (17.8)	−26.6 (41.1)	−50.4, -2.9
11-BS	5.3 (1.2)	−0.5 (3.0)	−3.7, 2.7	6.5 (1.9)	−2.2 (3.9)	−4.5, 0.03
Number of Pain tablets	27.3 (14.6)	−20.5 (16.4)	−37.7, -3.3	19.7 (27.1)	−5.3 (14.8)	−13.8, 3.3
SBP (mmHg)	138.0 (24.9)	0.0 (35.4)	−43.9, 43.9	127.1 (26.7)	−11.5 (24.5)	−29.0, 6.0
DBP (mmHg)	84.0 (5.5)	−4.0 (11.4)	−18.2, 10.2	76.5 (10.7)	−3.8 (14.3)	−14.1, 6.5
	Post-test mean score (SD)			Post-test mean score (SD)		
EARS (Average of 6 sessions)	21.4 (3.7)			17.0 (3.8)		

SBP=systolic blood pressure; DBP=diastolic blood pressure

### Participants’ experiences of the Good Back programme

More detailed explanation of themes, subthemes and narrative with participant’s quotes are given in Supplementary material.

**Table:** 

Themes	Positive perceptions of the programme	Good understanding of recommended self-management strategies	Adherence behaviour	Recommendations for programme improvement
Subthemes	Group structure of the programme was valued	Behaviour change was correctly understood as an ongoing process	Improvement of symptoms appeared to have the strongest influence on adherence behaviour	Shorter but ongoing programme sessions incorporating videos and print materials were suggested
	Improvements in symptoms positively influenced views of the programme	Exercise was correctly regarded as part of daily life	Expectation of symptom improvement was important for adherence	Spacious exercise/demonstration rooms in primary care centres was recommended
	Health professional-led intervention delivered in primary care centre was advocated		Adherence was facilitated by interesting practice sessions with self-help educational materials	Community involvement to reduce the stigma associated with exercise as treatment, and legitimise exercise for back pain management
	Enhancing participants’ knowledge of chronic low back pain via a collaborative communication style was appreciated		Non-adherence was related to contextual personal factors	

## Discussion

This is the first study exploring a novel evidence-based theory-informed biopsychosocial intervention for chronic LBP management in a rural African context. Overall, a good recruitment rate and intervention fidelity were observed. Good treatment uptake, retention and adherence to recommended self-management strategies, good acceptability and promising clinical outcomes were observed. These results provide a rationale for more rigorous intervention testing and potential implementation.

The recruitment rate was 100% (all the eligible participants wanted to participate). This is higher than 55–90% reported in the UK and USA (Morone *et al*., 2008; Bearne *et al*., 2011; Hunter *et al*., 2012). Limited access to effective musculoskeletal health care in rural Nigeria may have increased motivation to participate in this study. Although the overall recruitment rate was good, it is noteworthy that male participants were difficult to recruit which requires further investigation.

Convenient assignment rather than random assignment to the study arms could have reduced attrition rates by ensuring that the most motivated people participated in the self-management programme. The limitation is that it could have resulted in pre-existing differences that may in part explain the greater improvements in the self-management group. However, improvements in self-reported disability, fear avoidance beliefs and blood pressure which were balanced in the two groups is promising.

The acceptability of the programme was good, as all the participants preferred the programme over usual care. This is not surprising since the intervention was delivered at no cost with promising outcomes, whereas pain medication, the usual care for chronic LBP in rural Nigeria only has transient pain relief despite significant costs (Igwesi-Chidobe *et al*., 2017b).

The overall attendance at the programme was 83%, comparable with the 83.8% and 81% attendance at a mindfulness-based meditation programme for community dwelling older adults with chronic LBP in the USA (Morone *et al*., 2008), and exercise-based rehabilitation programme for chronic hip pain in the UK (Bearne *et al*., 2011). However, male participants had erratic attendance, which they associated with work. Delivering the intervention in work sites could be explored in future studies. Male participants might have also been uncomfortable attending a programme with a majority of women. An equal gender representation, or a group run specifically for men could be explored in future studies.

Exercise adherence was good possibly due to the use of a relevant theory, integrating CBT and motivational interviewing techniques, and assessing adherence between sessions and immediately after the programme. This is despite the community’s view of exercises as illegitimate treatment for chronic LBP which may inhibit long-term exercise behaviour change. Longer follow-up periods plus addressing negative community beliefs may be necessary in future studies in rural Nigeria.

Improvements in participants’ symptoms may have been the strongest determinant of both programme attendance and exercise adherence. This implies that future trials in this context must deliver interventions at a dose and duration sufficient to improve participants’ symptoms before follow-up. It is therefore unlikely that brief educational interventions without exercise demonstrations will be effective in rural Nigeria. Additionally, combined group and individual discussion sessions informed by CBT and motivational interviewing may have further increased autonomous motivation through a collaborative patient-centred communication style.

Reductions in self-reported disability, illness perceptions, fear avoidance beliefs, pain intensity and pain medication use concurs with the Leventhal’s self-regulatory model of illness cognitions (Leventhal *et al*., 1998). Thus, modification of back pain beliefs may have modified coping strategies and emotions and so influenced disability. However, effect sizes may have been moderated by unequal baseline scores for many outcomes.

Female participants had better outcomes with more precise estimates, except for pain medication use, for which they had a lower baseline value. This might be because male participants missed most of the group discussion sessions where psychosocial factors were specifically targeted. However, male participants’ lower baseline scores in the psychosocial factors and the very small sample size in this study may have tempered this finding, and hence needs further investigation in future studies.

Reduction in pain medication use, most of which were opioids such as tramadol, may be of public health importance. The long-term use of opioids increases cardiovascular risk and has limited use in chronic LBP (Carragee, 2005). Opioid medication dependence is a salient maladaptive coping strategy in this population with high prevalence of hypertension and diabetes comorbidity (Igwesi-Chidobe *et al*., 2017b).

Unexpected improvements in blood pressure, especially in the females, might be because targeting psychosocial factors and exercising may have modified pain experience and reduced stress (Truchon, 2001). Evidence suggests that psychosocial stress, increased by pain (Truchon, 2001), contributes to cardiovascular disease, and that stress reduction reduces blood pressure (Rainforth *et al*., 2007). However, the very small number of male participants means that this finding needs to be interpreted with caution. Blood pressure improvements may have also occurred through participants’ better adherence to antihypertensive drugs which may have been facilitated by the discussion sessions that contrasted medication use for self-management of chronic LBP from that for hypertension.

The Good Back programme seemed feasible and acceptable with promising clinical outcomes. It should be rigorously tested after incorporating the requested programme modifications. Exploration of necessary training and supervision needs of first line primary health care workers in rural Nigeria would be essential for refining this programme for a future randomised clinical trial and for possible implementation.

## Supplementary material

For supplementary material accompanying this paper visit https://doi.org/10.1017/S1463423619000070. click here to view supplementary material

## References

[ref1] Abdulraheem I (2007) Experience and perspectives of quality of health care in Nigerian rural community: an exploratory study. Nigerian Medical Practitioner 50, 48–53.

[ref2] Abegunde DO, Mathers CD, Adam T, Ortegon M Strong K (2007) The burden and costs of chronic diseases in low-income and middle-income countries. The Lancet 370, 1929–1938. 10.1016/S0140-6736(07)61696-1 18063029

[ref3] Barlow J, Wright C, Sheasby J, Turner A Hainsworth J (2002) Self-management approaches for people with chronic conditions: a review. Patient Education & Counseling 48, 177–187. 12401421 10.1016/s0738-3991(02)00032-0

[ref4] Barrowclough C, Haddock G, Wykes T, Beardmore R, Conrod P, Craig T, Davies L, Dunn G, Eisner E Lewis S (2010) Integrated motivational interviewing and cognitive behavioural therapy for people with psychosis and comorbid substance misuse: randomised controlled trial. BMJ 341, c6325. 21106618 10.1136/bmj.c6325 PMC2991241

[ref5] Bearne LM, Walsh NE, Jessep S Hurley MV (2011) Feasibility of an exercise‐based rehabilitation programme for chronic hip pain. Musculoskeletal Care 9, 160–168. 21695751 10.1002/msc.209

[ref6] Beaton DE, Bombardier C, Guillemin F Ferraz MB (2000) Guidelines for the process of cross-cultural adaptation of self-report measures. Spine 25, 3186–3191. 11124735 10.1097/00007632-200012150-00014

[ref7] Birabi BN, Dienye PO Ndukwu GU (2012) Prevalence of low back pain among peasant farmers in a rural community in South Nigeria. Rural & Remote Health 12, 1920. 22934910

[ref8] Broadbent E, Petrie KJ, Main J Weinman J (2006) The brief illness perception questionnaire. Journal of Psychosomatic Research 60, 631–637. 16731240 10.1016/j.jpsychores.2005.10.020

[ref9] Carragee EJ (2005) Persistent low back pain. New England Journal of Medicine 352, 1891–1898. 15872204 10.1056/NEJMcp042054

[ref10] Chen H.Y Boore J.R (2010) Translation and back‐translation in qualitative nursing research: methodological review. Journal of Clinical Nursing 19, 234–239. 19886874 10.1111/j.1365-2702.2009.02896.x

[ref11] Dean E Söderlund A (2015) What is the role of lifestyle behaviour change associated with non-communicable disease risk in managing musculoskeletal health conditions with special reference to chronic pain? BMC Musculoskeletal Disorders 16, 1. 25888381 10.1186/s12891-015-0545-y PMC4397667

[ref12] Downie A, Williams CM, Henschke N, Hancock MJ, Ostelo RW, DE Vet HC, Macaskill P, Irwig L, Van Tulder MW Koes BW (2013) Red flags to screen for malignancy and fracture in patients with low back pain: systematic review. BMJ: British Medical Journal 347, f7095. 24335669 10.1136/bmj.f7095 PMC3898572

[ref13] Du S, Yuan C, Xiao X, Chu J, Qiu Y Qian H (2011) Self-management programs for chronic musculoskeletal pain conditions: a systematic review and meta-analysis. Patient Education and Counseling 85, e299–e310. 21458196 10.1016/j.pec.2011.02.021

[ref14] Ehde DM, Dillworth TM Turner JA (2014) Cognitive-behavioral therapy for individuals with chronic pain: efficacy, innovations, and directions for research. American Psychologist 69, 153. 24547801 10.1037/a0035747

[ref15] Eldridge SM, Lancaster GA, Campbell MJ, Thabane L, Hopewell S, Coleman CL Bond CM (2016) Defining feasibility and pilot studies in preparation for randomised controlled trials: development of a conceptual framework. PLoS One 11, e0150205. 26978655 10.1371/journal.pone.0150205 PMC4792418

[ref16] Hawker GA, Mian S, Kendzerska T French M (2011) Measures of adult pain: Visual analog scale for pain (VAS pain), numeric rating scale for pain (NRS pain), McGill pain questionnaire (MPQ), Short‐Form McGill Pain Questionnaire (SF‐MPQ), Chronic Pain Grade Scale (CPGS), Short Form‐36 Bodily Pain Scale (SF‐36 BPS), and measure of intermittent and constant osteoarthritis pain (ICOAP). Arthritis Care & Research 63, S240–S252. 22588748 10.1002/acr.20543

[ref17] Heymans MW, VAN Tulder MW, Esmail R, Bombardier C Koes BW (2005) Back schools for nonspecific low back pain: a systematic review within the framework of the Cochrane Collaboration Back Review Group. Spine 30, 2153–2163. 16205340 10.1097/01.brs.0000182227.33627.15

[ref18] Hoy D, Brooks P, Blyth F Buchbinder R (2010) The epidemiology of low back pain. Best Practice & Research Clinical Rheumatology 24, 769–781. 21665125 10.1016/j.berh.2010.10.002

[ref19] Hunter RF, Mcdonough SM, Bradbury I, Liddle SD, Walsh DM, Dhamija S, Glasgow P, Gormley G, Mccann SM Park J (2012) Exercise and auricular acupuncture for chronic low-back pain: a feasibility randomized-controlled trial. The Clinical Journal of Pain 28, 259–267. 21753728 10.1097/AJP.0b013e3182274018

[ref20] Igwesi-Chidobe C (2012) Obstacles to obtaining optimal physiotherapy services in a rural community in southeastern Nigeria. Rehabilitation Research and Practice. 2012. 10.1155/2012/909675 PMC3437668 22973517

[ref21] Igwesi-Chidobe CN, Coker B, Onwasigwe CN, Sorinola IO Godfrey EL (2017a) Biopsychosocial factors associated with chronic low back pain disability in rural Nigeria: a population-based cross-sectional study. BMJ Global Health 2, e000284. 10.1136/bmjgh-2017-000284 PMC5717944 29225944

[ref22] Igwesi-Chidobe C, Kengne AP, Sorinola I Godfrey E (2018) Physical activity containing behavioural interventions for adults living with modifiable chronic non-communicable diseases in Africa: a systematic mixed-studies review. International Health. 10.1093/inthealth/ihy013 29554307

[ref23] Igwesi-Chidobe C, Kitchen S, Sorinola I Godfrey E (2015) Qualitative exploration of non-specific chronic low back pain in rural Nigeria: towards evidence-informed physiotherapy practice. Physiotherapy 101, e636–e637.

[ref24] Igwesi-Chidobe CN, Kitchen S, Sorinola IO Godfrey EL (2017b) “A life of living death”: the experiences of people living with chronic low back pain in rural Nigeria. Disability and Rehabilitation 39, 779–790. 27111492 10.3109/09638288.2016.1161844

[ref25] Igwesi-Chidobe CN, Obiekwe C, Sorinola IO Godfrey EL (2017c) Assessing self-reported disability in a low-literate population with chronic low back pain: cross-cultural adaptation and psychometric testing of Igbo Roland Morris disability questionnaire. Disability and Rehabilitation 1–10. 10.1080/09638288.2017.1416185 29239235

[ref26] Jones SH, Barrowclough C, Allott R, Day C, Earnshaw P Wilson I (2011) Integrated motivational interviewing and cognitive–behavioural therapy for bipolar disorder with comorbid substance use. Clinical Psychology & Psychotherapy 18, 426–437. 21898672 10.1002/cpp.783

[ref27] Lakens D (2013) Calculating and reporting effect sizes to facilitate cumulative science: a practical primer for t-tests and ANOVAs. Frontiers in Psychology 4, 1–12. 24324449 10.3389/fpsyg.2013.00863 PMC3840331

[ref28] Lawn S Schoo A (2010) Supporting self-management of chronic health conditions: common approaches. Patient Education & Counseling 80, 205–211. 19931372 10.1016/j.pec.2009.10.006

[ref29] Leventhal H, Leventhal EA Contrada RJ (1998) Self-regulation, health, and behavior: a perceptual-cognitive approach. Psychology and Health 13, 717–733.

[ref30] Liddle SD, Baxter GD Gracey JH (2004) Exercise and chronic low back pain: what works? Pain 107, 176–190. 14715404 10.1016/j.pain.2003.10.017

[ref31] Mcnee P, Shambrook J, Harris EC, Kim M, Sampson M, Palmer KT Coggon D (2011) Predictors of long-term pain and disability in patients with low back pain investigated by magnetic resonance imaging: a longitudinal study. BMC Musculoskeletal Disorders 12, 234. 21999666 10.1186/1471-2474-12-234 PMC3219563

[ref32] Morone NE, Greco CM Weiner DK (2008) Mindfulness meditation for the treatment of chronic low back pain in older adults: a randomized controlled pilot study. Pain 134, 310–319. 17544212 10.1016/j.pain.2007.04.038 PMC2254507

[ref33] Newman-Beinart NA, Norton S, Dowling D, Gavriloff D, Vari C, Weinman JA Godfrey EL (2017) The development and initial psychometric evaluation of a measure assessing adherence to prescribed exercise: the Exercise Adherence Rating Scale (EARS). Physiotherapy 103, 180–185. 27913064 10.1016/j.physio.2016.11.001

[ref34] Nice (2009) Low back pain: early management of persistent non-specific low back pain. *In* Care N. C. C. F. P, editor London: NHS. 20704057

[ref35] Pincus T, Anwar S, Mccracken L, Mcgregor A, Graham L, Collinson M Farrin AJ (2013) Testing the credibility, feasibility and acceptability of an optimised behavioural intervention (OBI) for avoidant chronic low back pain patients: protocol for a randomised feasibility study. Trials 14, 172. 23764140 10.1186/1745-6215-14-172 PMC3691616

[ref36] Pincus T, Anwar S, Mccracken LM, Mcgregor A, Graham L, Collinson M, Mcbeth J, Watson P, Morley S Henderson J (2015) Delivering an Optimised Behavioural Intervention (OBI) to people with low back pain with high psychological risk; results and lessons learnt from a feasibility randomised controlled trial of Contextual Cognitive Behavioural Therapy (CCBT) vs. Physiotherapy. BMC Musculoskeletal Disorders 16, 1. 26076755 10.1186/s12891-015-0594-2 PMC4468803

[ref37] Rainforth MV, Schneider RH, Nidich SI, Gaylord-King C, Salerno J. W Anderson JW (2007) Stress reduction programs in patients with elevated blood pressure: a systematic review and meta-analysis. Current Hypertension Reports 9, 520–528. 18350109 10.1007/s11906-007-0094-3 PMC2268875

[ref39] Steenstra I, Verbeek J, Heymans M Bongers P (2005) Prognostic factors for duration of sick leave in patients sick listed with acute low back pain: a systematic review of the literature. Occupational and Environmental Medicine 62, 851–860. 16299094 10.1136/oem.2004.015842 PMC1740930

[ref40] Strand LI, Moe-Nilssen R Ljunggren AE (2002) Back performance scale for the assessment of mobility-related activities in people with back pain. Physical Therapy 82, 1213–1223. 12444880

[ref41] Thabane L, Hopewell S, Lancaster GA, Bond CM, Coleman CL, Campbell MJ Eldridge SM (2016) Methods and processes for development of a CONSORT extension for reporting pilot randomized controlled trials. Pilot and Feasibility Studies 2, 1. 27965844 10.1186/s40814-016-0065-z PMC5153862

[ref42] Truchon M (2001) Determinants of chronic disability related to low back pain: towards an integrative biopsychosocial model. Disability and Rehabilitation 23, 758–767. 11762878 10.1080/09638280110061744

[ref43] Van Middelkoop M, Rubinstein SM, Kuijpers T, Verhagen AP, Ostelo R, Koes BW Van Tulder MW (2011) A systematic review on the effectiveness of physical and rehabilitation interventions for chronic non-specific low back pain. European Spine Journal 20, 19–39. 20640863 10.1007/s00586-010-1518-3 PMC3036018

[ref44] Waddell G, Newton M, Henderson I, Somerville D Main CJ (1993) A Fear-Avoidance Beliefs Questionnaire (FABQ) and the role of fear-avoidance beliefs in chronic low back pain and disability. Pain 52, 157–168. 8455963 10.1016/0304-3959(93)90127-B

[ref45] Warsi A, Lavalley MP, Wang PS, Avorn J Solomon DH (2003) Arthritis self‐management education programs: a meta‐analysis of the effect on pain and disability. Arthritis & Rheumatism 48, 2207–2213. 12905474 10.1002/art.11210

