# Supplementary material for: Community-based self-management of chronic low back pain in a rural African primary care setting: a feasibility study
Source: Prim Health Care Res Dev. 2019 Apr 29;20:e45. doi: 10.1017/S1463423619000070 (PMC6536765; doi:10.1017/S1463423619000070)
Supplement: Supplementary file 1 [file S1463423619000070sup001.docx]

*Supplemental material*

**Participants’ experiences of the Good Back programme**

1. **Positive perceptions of the programme**

**Group structure of the programme was valued**

All but one respondent that participated in the programme preferred a group-based programme incorporating exercise demonstration and postural hygiene for functional activities. They reported that these groups facilitated social support, collaborative learning and fun activities.

*‘It is better group-based because it enables you to see other people having the condition and learn from other people’ (P1).*

The only participant who did not like a group format was male, and attended only half of one session (before phase 4) out of the six sessions of the programme. He was not aware of the individual discussion sessions in the programme. He was also by far the youngest participant.

*‘I didn’t like it being group-based because I am a shy person’ (P2).*

**Improvements in symptoms positively influenced views of the programme**

Eight of the participants reported that their main reasons for liking the programme was because it reduced their back pain, increased their functional ability and sense of well-being. One of these eight participants reported that he no longer depended on drugs to perform his daily activities, and likened the effects of the programme to that obtained from analgesic drugs.

*‘…in fact, it is like I am taking drugs because after the exercises I become fit to do my work (P13).*

**Health professional-led intervention delivered in primary care centre was advocated**

All participants that attended the programme preferred the programme to be delivered by health professionals in primary care centres. They believed that only health professionals had the pre-requisite knowledge, skills and experience to deliver the programme. Participants believed that there were two broad categories of health professionals: ‘doctors’ and ‘nurses’. They regarded the lead author, a physiotherapist, as a ‘doctor’, and the community health workers who are the first line primary care workers in rural Nigeria as ‘nurses’. Two of these participants advocated for any health professional with expertise in exercise therapy or who has chronic LBP because they believed such people will be more competent in delivering the intervention.

*‘a health professional trained in exercise treatment’ (P12); ‘a health professional who also has back pain’ (P6).*

All but one of the participants did not want a lay person delivering the intervention due to a perceived lack of expertise and experience. Programmes delivered in primary care centres were preferred for legitimacy and accessibility.

*‘Health centre is best. Going to the field or community centre will make the programme not to be perceived as serious (P9).*

**Enhancing participants’ knowledge of chronic low back pain via a collaborative communication style was appreciated**

Three participants acknowledged and appreciated improvements in their knowledge of chronic LBP due to the collaborative communication style that provided the opportunity to converse with and question the health professional. This style of communication enabled participants to present and defend their ideas, exchange varied beliefs and be actively engaged during the sessions.

*What I liked best about the treatment is how you asked us questions and we answered them which helped us understand things better (P6).*

This improvement in the knowledge about chronic LBP and the management strategies was reported as the underlying factor that reduced drug dependence in one participant.

*‘I used to take drugs all the time…, but now I understand better and use exercises for my back pain’ (P5).*

1. **Good understanding of recommended self-management strategies**

**Behavior change was correctly understood as an ongoing process**

All participants that attended the programme understood that the recommended self-management strategies were meant to be permanent in their lives.

**Exercise was correctly regarded as part of daily life**

All but two participants understood that exercises should be a part of their daily life. One of these participants with a poor understanding was the one that attended only half of one session (before phase 4) out of the six programme sessions.

1. **Adherence behaviour**

**Improvement of symptoms appeared to have the strongest influence on adherence behaviour**

The participants’ most cited reason for good adherence was because they experienced a reduction in back pain symptoms as well as symptoms they associated with their co-morbid chronic health conditions, such as hypertension and diabetes. They reported improved feelings of well-being, functional ability, hopefulness and sleep. Eight participants stated this as their main reason.

*‘I did the exercises because things I could not do before, I can do them now’ (P11).*

**Expectation of symptom improvement was important for adherence**

Four participants reported that their reasons for carrying out the recommended self-management strategies were due to expectations that these strategies would be helpful for their chronic pain, co-morbid chronic conditions and general health in the long term. However, one of these four participants attended only half of one session out of the six sessions of the programme and still had expectations of being cured by the programme.

*‘I was doing the exercises because I need to get healed’ (P2).*

**Adherence was facilitated by interesting practice sessions with self-help educational materials**

Three participants reported that they did the recommended self-management strategies due to the training and the self-help educational materials they received during the practice sessions which increased their understanding of what was required. The engaging practice sessions made them enjoy the programme and increased their desire to maintain the recommended strategies.

*‘I was doing the exercises because I understood them and started enjoying them…the sessions were fun’ (P7).*

**Non-adherence was related to contextual personal factors**

A few participants who had poor exercise adherence appeared to be influenced mainly by the presence of chronic co-morbid conditions such as hypertension, or younger age or poor attendance at the programme. One obese participant with hypertension and diabetes did not do many of the exercises because she believed that exercises were dangerous for her. This perception was in line with her understanding of her doctor’s recommendations. She believed that performing exercises would increase her symptoms of hypertension.

*‘I do not do all my exercises because of my dizziness due to my hypertension’ (P8)*

The youngest participant, a male student, reported that his poor attendance was because of lack of time. Four participants had their adherence temporarily halted by acute conditions including typhoid fever, malaria and domestic accident.

1. **Recommendations for programme improvement**

**Shorter but ongoing programme sessions incorporating videos and print materials were suggested**

All participants recommended that the programme should be ongoing and permanent in the primary centre, and done on specific days. Three participants requested that the duration of each session be shortened to give them time to collect their children from school. One participant suggested the inclusion of videos demonstrating the exercises and postural hygiene in the self-help educational materials. They reported that these could simulate the group practice sessions, improve understanding of the recommended self-management strategies and enhance long term adherence.

*‘Including a video showing how to do the exercises which we can use at home …so that we can continue using it when the programme is over’ (P10).*

**Spacious exercise/demonstration rooms in primary care centres was recommended**

Two participants suggested that bigger rooms should be provided in the primary care centres to improve the group exercise sessions.

*‘a bigger place to allow more movements during the warm up exercises’ (P1)*

**Community involvement to reduce the stigma associated with exercise as treatment, and legitimise exercise for back pain management**

All participants reported being mocked and laughed at by community members because they were performing exercise which was not regarded as legitimate treatment in rural Nigeria.

*‘they were saying, where are the drugs you were given for your pain? then they said, you are just going to the health centre to play… ‘(P10).*

Participants felt that changes at the community level would enhance the impact of the programme. For instance, three participants felt that increasing awareness about the benefits of exercise would enhance the programme’s effectiveness, and reduce the stigma associated with doing exercises as treatment. One participant suggested that mass media could be a potentially effective source of enlightenment about exercise as treatment for back pain.

*‘Raising awareness so that more people know about exercises’ (P10).*
